# The promotion of angiogenesis induced by three-dimensional porous beta-tricalcium phosphate scaffold with different interconnection sizes via activation of PI3K/Akt pathways

**DOI:** 10.1038/srep09409

**Published:** 2015-03-23

**Authors:** Xin Xiao, Wei Wang, Dong Liu, Haoqiang Zhang, Peng Gao, Lei Geng, Yulin Yuan, Jianxi Lu, Zhen Wang

**Affiliations:** 1 Department of Orthopedics, Xijing Hospital, Forth Military Medical University, Xi'an, Shaanxi, PR China; 2 The State Key Laboratory of Cancer Biology, Department of Immunology, Forth Military Medical University, Xi'an, Shaanxi, PR China; 3 Shanghai Bio-lu Biomaterials Co., Ltd., Shanghai, P.R. China

## Abstract

The porous architectural characteristics of biomaterials play an important role in scaffold revascularization. However, no consensus exists regarding optimal interconnection sizes for vascularization and its scaffold bioperformance with different interconnection sizes. Therefore, a series of disk-type beta-tricalcium phosphates with the same pore sizes and variable interconnections were produced to evaluate how the interconnection size influenced biomaterial vascularization *in vitro* and *in vivo*. We incubated human umbilical vein endothelial cells on scaffolds with interconnections of various sizes. Results showed that scaffolds with a 150 μm interconnection size ameliorated endothelial cell function evidenced by promoting cell adhesion and migration, increasing cell proliferation and enhancing expression of platelet-endothelial cell adhesion molecules and vascular endothelial growth factor. *In vivo* study was performed on rabbit implanted with scaffolds into the bone defect on femoral condyles. Implantation with scaffolds with 150 μm interconnection size significantly improved neovascularization as shown by micro-CT as compared to scaffolds with 100 and 120 μm interconnection sizes. Moreover, the aforementioned positive effects were abolished by blocking PI3K/Akt/eNOS pathway with LY-294002. Our study explicitly demonstrates that the scaffold with 150 μm interconnection size improves neovascularization via the PI3K/Akt pathway and provides a target for biomaterial inner structure modification to attain improved clinical performance in implant vascularization.

Porous, three-dimensional biomaterials have been used extensively as scaffolds in the field of tissue engineering. Most bodily tissues rely on blood vessels to supply the individual cells with nutrients and oxygen. For a tissue to grow beyond 100–200 mm (the diffusion limit of oxygen), new blood-vessel formation is required^1^, and this is also true for tissue-engineered constructs. To increase tissue-engineered constructs vascularization, several approaches have been employed such as *ex vivo*-culturing of scaffolds with endothelial cells alone or combined with fibroblasts, the addition of angiogenic growth factors, and provision of a vascularized tissue flap^2^,^3^,^4^,^5^,^6^,^7^,^8^. However, without appropriate porous structures for blood vessel formation in scaffolds, the effectiveness of these approaches on angiogenesis in transplants will be limited^9^,^10^,^11^,^12^,^13^,^14^,^15^,^16^. Several studies have shown that appropriate internal scaffold structure can allow the nutrients to infiltrate and provide pathways for new blood vessel ingrowth^11^,^12^,^14^,^15^,^16^,^17^,^18^. The pore size and interconnections between macropores are porous scaffold structure parameters and are two essential factors for blood vessel growth^12^,^13^,^14^,^15^,^16^. Although pore size has a strong impact on vascularization, pore interconnection is more critical for biomaterial scaffolds^12^,^13^,^14^. An incomplete pore interconnection could constrain the overall biological system, limiting blood vessel invasion. Although few literary reports indicate interconnection has a beneficial effect on vascularization^12^,^14^,^16^,^17^, it is still not completely clear how this parameter influences the blood vessel formation process. Moreover, no consensus exists regarding the optimal porous structure for vascularization. Therefore, biomaterials need appropriate interconnection pathways to allow the blood vessels to infiltrate the scaffold.

To evaluate the effect of a single structural parameter on biomaterials' biological properties, other parameters had to be kept identical. Therefore, an accurate porous structure control is required to investigate interconnection sizes' effects on biomaterials vascularization, and this is a lofty challenge in the fabrication of the porous bioceramics. Conventional scaffold fabrication techniques including gas foaming, phase separation, freeze drying and particulate leaching are widely used to produce 3D scaffolds for tissue-engineering applications. Although the pore shape and size can be varied by changing their parameters, the resulting pore organization is random. This can lead to pore pathways that are only partially connected and that follow contorted routes. Accordingly, few studies specifically and systematically investigated the role of the interconnection size in neovascularization in porous biomaterials. Furthermore, most of these conclusions were based on studies using materials with random pore geometry, wide porosity range, and variously sized pores with smaller interconnection or even without an interconnection pathway due to inaccurately controlling the porous scaffold structure^11^,^12^,^14^,^16^,^17^. Therefore, a technique is needed that can fabricate the bioceramic scaffolds with high porosity and accurately controlled porous structure.

Beta-tricalcium phosphate (β-TCP) has good biocompatibility and osteoconductivity in both animal experiments and clinical settings^19^,^20^,^21^ and its composition is near the bone. Moreover, its resorbability allows a gradual biological degradation over a period of time and a progressive replacement by the natural host tissue^22^. This property imparts significant advantages onto β-TCP compared to other biomedical materials, which are not resorbed and replaced by natural bone^23^. Our previous study showed that pure β-TCP was completely resorbed at skeletal sites over 24 weeks^14^. Its degradation rate can meet bone repair requirements.

Thus, in this study, we developed β-TCP with accurately controlled pore size and interconnections using slip casting shaping and polymethylmethacrylate (PMMA) as a porogen agent. There was no significant difference between porous structure parameters except interconnection size in these scaffolds. Both *in vitro* and *in vivo* studies were designed to investigate interconnections' effects on human umbilical vein endothelial cells (HUVECs) which are important in angiogenesis. We investigated HUVEC proliferation, migration and cell-specific marker expression in response to different porous structures, focusing on the related protein level mechanisms. Furthermore, a femur defect rabbit model was applied for transplanting porous β-TCP scaffolds, and the *in vivo* neoangiogenesis was investigated. Finally, we determined the optimal porous structure for vascularization.

## Results

### The structural characterization of porous β-TCP bioceramics

**Figure 1:**
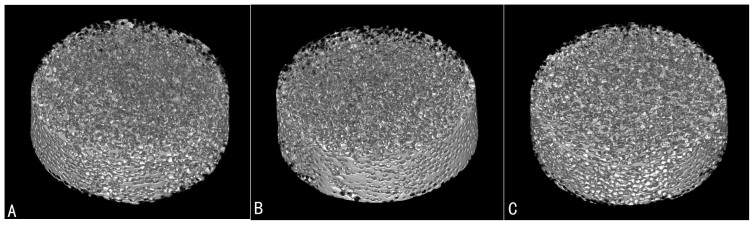
The 3D images of porous β-TCP scaffolds with different interconnection sizes of (A) 100 μm, (B) 120 μm, (C) 150 μm and the same pore sizes (300-400 μm) by Micro-CT.

**Figure 2:**
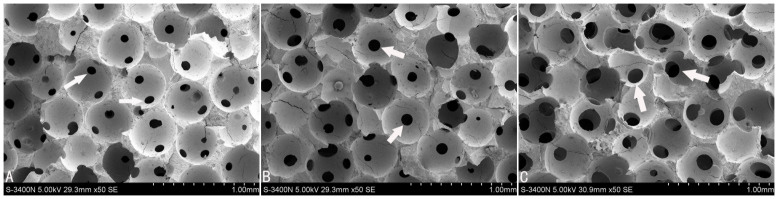
The scanning electron micrographs show that porous β-TCP bioceramics with different interconnection sizes of (A) 100 μm, (B) 120 μm, (C) 150 μm and the same pore sizes (300-400 μm). Scale bars: 1.00 mm. White arrow indicates interconnection between macropores.

We prepared a series of scaffolds with the same 300–400 μm macropore sizes but with different interconnection diameters ranging from 100 to 150 μm. All β-TCP scaffolds represented similar macrostructures, consisting of well-interconnected and regular spherical macropores, but had different interconnection diameters between macropores. Figure 1 showed the 3D images of porous β-TCP with different interconnecton sizes of 100, 120 and 150 μm by micro-CT. Identical sphere-shaped macropores were seen in every porous β-TCP bioceramic and at least one small and identical pore as interconnection within every macropore was observed (Fig. 2). The measured mean interconnection size was also accurately controlled in the theoretical scope (Table 1). The estimated total porosity of all scaffolds was above 70% suitable for tissue engineering^17^. Also, increased interconnection size resulted in an increase in scaffold porosity. The relevant data are summarized in Table 1.

### Cell morphologies on the β-TCP scaffold

**Figure 3:**
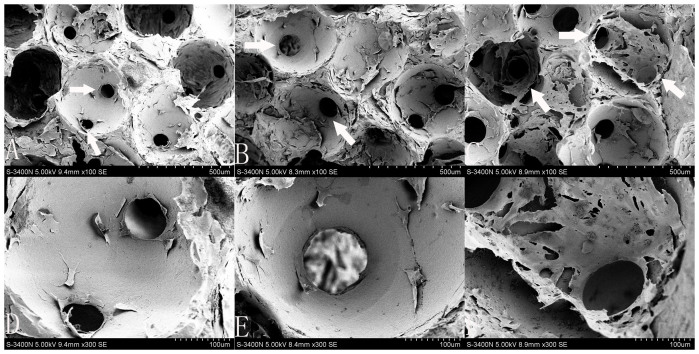
SEM images of HUVECs growing on the β-TCP scaffolds after 7 days of culture. A and D: 100 μm, B and E: 120 μm, C and F: 150 μm. D, E and F show cells around connecting pores at a higher magnification. The 150 μm interconnection sized scaffolds are covered with more dense and thick cell layers than those at 100 and 120 μm. White arrow indicates connecting pores between macropores.

In this study, SEM was used to investigate HUVEC morphology on the β-TCP scaffolds after 7 days of incubation (Fig. 3). The cells cultured on scaffolds with interconnection sizes of 100 and 120 μm formed a flattened endothelial cell layer. At 150 μm, the endothelial cells appeared more large-pieced and elongated with several cytoplasmic digitations. The large-pieced cells covered the majority of the scaffold's surface and inner macropores, and a dense and thick endothelial layer formed (Fig. 3C). Interestingly, more endothelial cells were gathered around 150 μm connecting holes and grew into the inner part of scaffold (Fig. 3F). These results indicated scaffolds with 150 μm interconnection size were beneficial for cell adhesion.

### Proliferation of HUVECs cultured on scaffold

**Figure 4:**
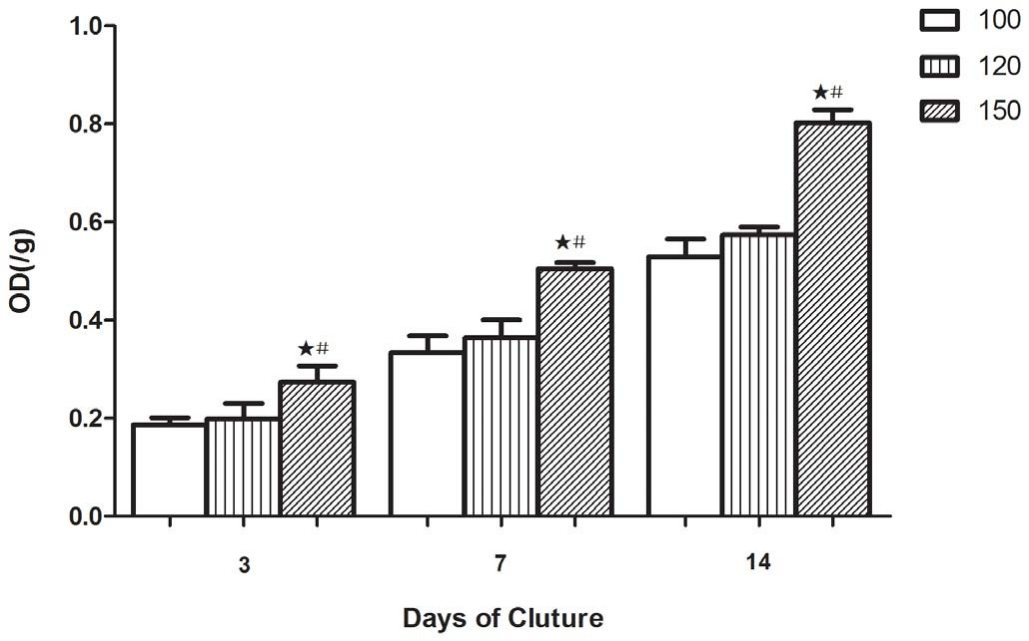
Cell viability of the cell/scaffold constructs over 14 days of culture. There was a continuing increase on all three kinds of scaffolds. There was a significant difference between the 150 μm interconnection sized scaffolds and the other two kinds of scaffolds at the same time point (n = 3; #, the difference attained a statistically significant increase compared to the 120 μm group, P < 0.05; 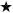, the difference attained a statistically significant increase compared to the 100 μm group, P < 0.05).

The cell viability on scaffolds with different interconnection sizes after 3, 7 and 14 days of incubation was evaluated by the MTT assay (Fig. 4). We observed increased cell viability over time in all three scaffold types. At all time points, cells cultured on 120 μm displayed slightly higher viability than that on 100 μm but there was no statistical difference (p > 0.05). At day 3, the 150 μm treatment increased cell viability while it was much higher on 120 μm (0.20 ± 0.03/g, p < 0.05) than that on 100 μm (0.18 ± 0.02/g, p < 0.05). At days 7 and 14, the differences between interconnection sizes of 150 μm and 100 or 120 μm were much more obvious, especially after incubation for 14 days (0.53 ± 0.03/g for 100 μm and 0.57 ± 0.02/g for 120 μm, both p < 0.05).

**Figure 5:**
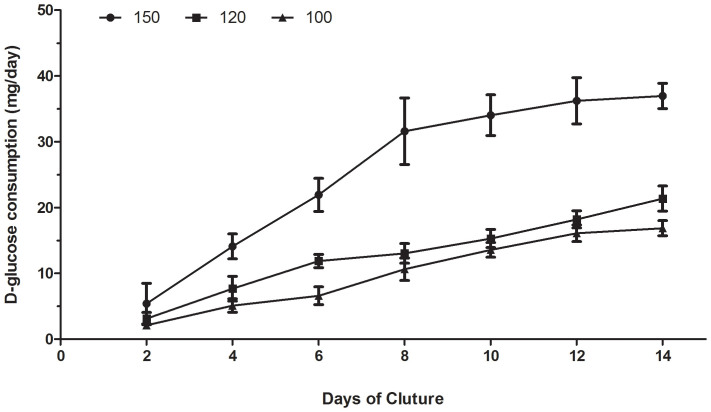
The daily glucose consumption of the cell/scaffold constructs with different interconnection sizes. The consumption increased gradually in all three groups. The increase was much greater in the 150 μm group compared to the 100 and 120 μm groups (p < 0.05).

The daily D-glucose consumption assay results were depicted in Fig. 5. The increased daily glucose consumption over time during the culture was similar to the increased cell viability in scaffolds with interconnection sizes 100, 120 and 150 μm. The increase was much greater under a 150 μm culture compared to a 100 or 120 μm culture at all time points (p < 0.05), except for the second day. Under the 150 μm treatment, there was a rapid increase in the first 7 days and then consumption increased slowly in the second 7 days of culture. Under the 100 or 120 μm treatments, the consumption relatively gradually increased over the entire culture period.

### Effect of different scaffolds on HUVEC function

**Figure 6:**
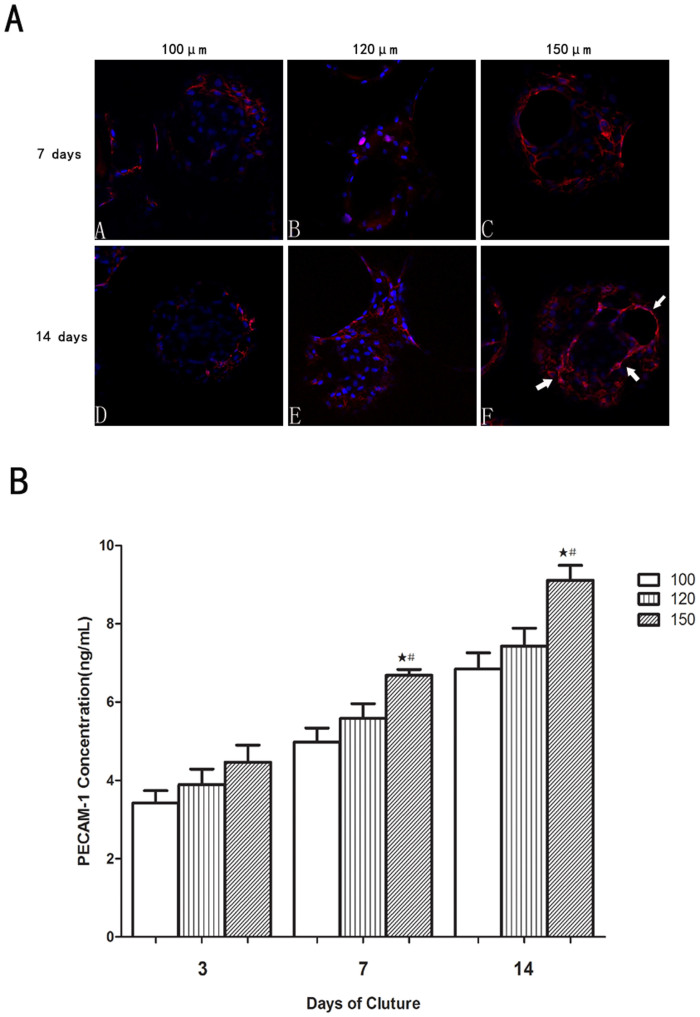
(A) CLSM images showing expression of endothelial marker, PECAM-1 (CD31), by HUVECs seeded on β-TCP scaffolds with different interconnection sizes at day 7and day 14. Confocal images through a z-stack demonstrated CD31expression. White arrow shows that the network of HUVEC was clearly observed in scaffolds with interconnection sizes of 150 μm. CD31 is in red (labeled with Alexa Fluor 594), and nuclei arein blue (labeled with DAPI). (B) The effect of scaffolds with different interconnection sizes on PECAM-1 expression at different culture times. (n = 3; #, the difference attained a statistically significant increase compared to the 120 μm group, P < 0.05; 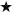, the difference attained a statistically significant increase compared to the 100 μm group, P < 0.05).

To understand the interactions of different interconnection sizes with HUVECs, PECAM-1 expression, NO production and VEGF secretion were measured. PECAM-1 expression of HUVECs on these scaffolds in different cultured times was determined using ELISA (Fig. 6B). As shown, the PECAM-1 concentration in the 150 μm group was significantly better than the 100 μm and 120 μm groups, except for day 3. The expression of PECAM-1 in 150 μm group displayed a slightly higher concentration than in the 100 μm and 120 μm groups, but there was no statistical difference (p > 0.05) after incubating for 3 days. After 7 days of culture, statistical difference was found between the 150 μm and 100 or 120 μm groups. At day 14, the PECAM-1 concentration in 150 μm group was markedly increased by about 33% and 23% compared to the 100 and 120 μm groups, respectively (6.85 ± 0.4 ng/ml for 100 μm and 7.43 ± 0.4 ng/ml for 120 μm, both p < 0.05).

Similar results were also seen in an immunofluorescent analysis of PECAM-1 expression. The immunofluorescent images showed PECAM-1 expression at the cell-cell interface after 14 days of culture (Fig. 6A). For all groups, PECAM-1 expression increased with culture time. What's more, in 150 μm group, PECAM-1 expression of HUVECs became more pronounced, and cells were observed forming networks after 14 days. In the 100 and 120 μm groups, these network structures were not apparent, perhanps due to the lower proportion of HUVECs present.

**Figure 7:**
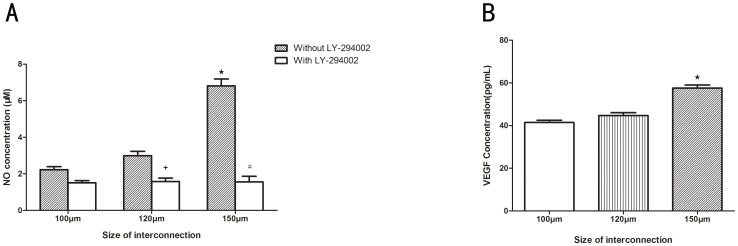
NO production (A) and VEGF secretion (B) of HUVECs cultured on scaffolds with different interconnection sizes for 7 days. (n = 3, 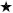 mean the difference attained a statistically significant increase compared to 120 μm group, P < 0.05; # mean the difference attained a statistically significant decrease compared to 150 μm group incubated without LY-294002, P < 0.05; + mean the difference attained a statistically significant decrease compared to 120 μm group incubated without LY-294002, P < 0.05).

As indicated in Fig. 7, significant increase of NO concentration (Fig. 7A) (p < 0.05) was observed when incubated on a scaffold with 150 μm interconnection size for 7 days compared to the 100 μm and 120 μm sizes. The NO concentration in 150 μm group incubated with LY-294002 (1.56 ± 0.43 μM/ml) was markedly depressed by about 77% compared with that incubated without LY-294002 (p < 0.05). Similar results were also seen in 100 and 120 μm groups (Fig. 7A). The secretion of VEGF was determined using ELISA. Fig. 7B shows that VEGF secretion was obviously enhanced in HUVECs cultured on a scaffold with a 150 μm interconnection size compared to the 100 and 120 μm sizes (p < 0.05). All these results demonstrated that the scaffold with an interconnection size of 150 μm possesses high ability to induce angiogenesis *in vitro*.

### Visualization of F-actin cell filaments

**Figure 8:**
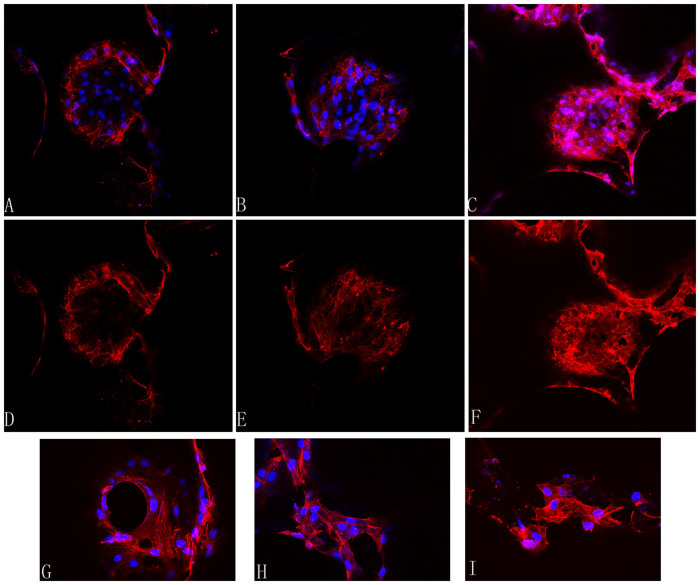
Cytoskeletal organization of cells grown on the β-TCP scaffolds at day 7, as observed under a CLSM and demonstrated by rhodamine phalloidin/DAPI staining for Factin/cell nuclei (A-I). F-actin fiber distributed on the scaffolds with interconnection sizes 100 μm (A and D), 120 μm (B and E) and 150 μm (C and F). Much more expression of F-actin fiber is observed in the 150 μm group (C, F). G, H and I show F-actin staining of HUVECs at a higher magnification.

HUVECs were double-stained with Rhodamine-phalloidin and DAPI to investigate the cytoskeletal organization and cell density after 7 days of incubation on different scaffolds (Fig. 8). Images showed that after incubating on a scaffold with an interconnection size of 150 μm, endothelial cells exhibited substantial cell area and great density with well-formed cytoskeletal organization (Fig. 8A–C). We saw clearly that, in the 100 μm group, the actin bundles only existed in the edges of macropores, while a small amount of actin fibers existed in the inner part of macropores in the 120 μm group. Conversely, staining the group of HUVECs seeded on 150 μm indicated a homogenous distribution of actin fibers and cell filament elongation on the whole macropore (Fig. 8D–F). Additionally, comparing the rhodamine phalloidin staining images in Fig. 8D–F, the cells seeded on 150 μm scaffolds had many more actin fibers than the 100 and 120 μm scaffolds. Contacting zones or overlapping growths were observed in Fig. 8F and Fig. 8G–I show the F-actin staining of HUVECs at a higher magnification, respectively. Obvious actin fiber bundles could be observed in the cytoplasm.

### The activation of eNOS through Akt dependent pathways in HUVECs

**Figure 9:**
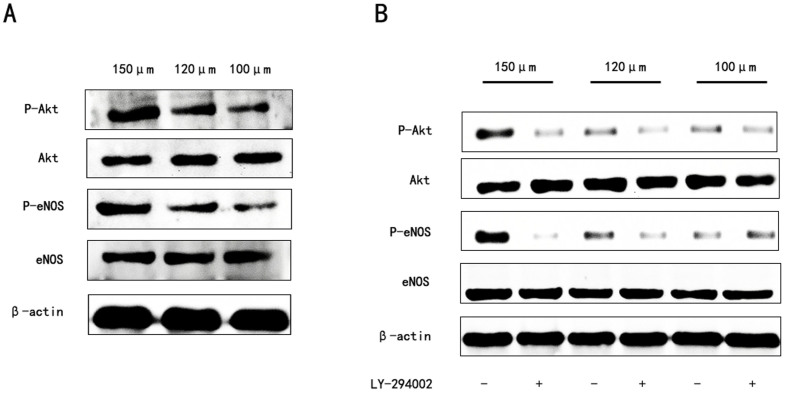
Activation of the PI3k/Akt pathway in HUVECs. (A) The expression profile of key proteins in the PI3k/Akt pathway in HUVECs cultured on scaffolds with different interconnection sizes for 7 days. (B) HUVECs were cultured on scaffolds with different interconnection sizes with or without 30 μM MLY-294002, respectively.

To determine if scaffold interconnection size could mediate eNOS activation through Akt dependent pathways, the expression of key molecules in the Akt signaling pathways were examined by western blot analysis. As depicted in Fig. 9A, compared to the scaffolds with interconnection sizes of 100 and 120 μm, the scaffold with an interconnection size of 150 μm generated a significant increase in the protein levels of p-eNOS. Meanwhile, phosphorylation of Akt also significantly enhanced when HUVECs were seeded on scaffolds with an interconnection size of 150 μm for 7 days. Moreover, phosphatidylinositol 3-kinase (PI3K) inhibitor LY-294002 blocked the expression of p-eNOS after 7 days (Fig. 9B).

### Effect of different scaffolds on neovascularization

**Figure 10:**
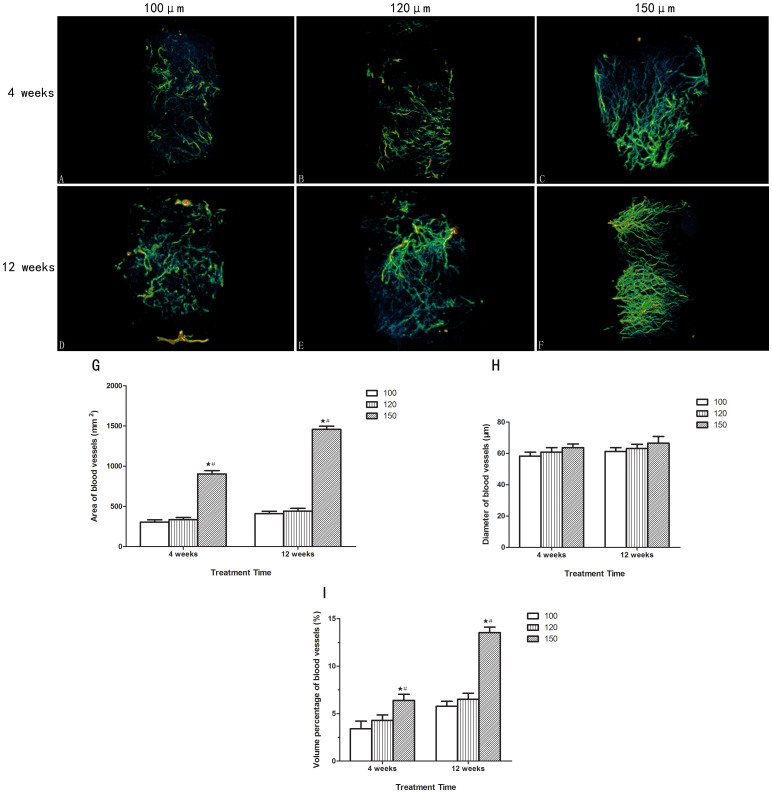
Evaluation of neovascularization of the implants with Micro-CT at 4 and 12 weeks after implantation. (A-F) Typical Micro-CT 3D reconstruction images of new blood vessel ingrowth within scaffolds with different interconnection sizes at 4 and 12 weeks after implantation. (A and D): 100 μm; B and E: 120 μm; (C and F): 150 μm. (G-I) Quantitative analysis results from Micro-CT evaluation. (#, the difference attained a statistically significant increase compared to the 120 μm group, P < 0.05; 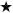, the difference attained a statistically significant increase compared to the 100 μm group, P < 0.05).

Typically reconstructed 3D stereoscopic pictures of scaffolds and newly formed blood vessels were obtained from a local Micro-CT scan and new blood vessels were analyzed at 4 and 12 weeks after implantation (Fig. 10).

#### The area of blood vessels formed in scaffolds

Figure 10G showed the area of blood vessels in scaffolds with different interconnection sizes. The blood vessel areas increased with increasing interconnection size. No significant difference in the blood vessels areas was shown between scaffolds with interconnection sizes of 100 and 120 μm at 4 and 12 weeks (p > 0.05). At 4 weeks, the samples with a 150 μm interconnection size had significantly larger blood vessel areas than samples with interconnection sizes of 100 and 120 μm (p < 0.05). At 12 weeks, scaffolds with a 150 μm interconnection size exhibited more favorable new blood vessels in the implant (Fig. 10C, F), and the blood vessel area (mm^2^) in 150 μm was markedly larger than that in 100 μm and 120 μm (p < 0.05). Therefore, interconnection size had a significant effect on the area of blood vessels formed.

#### The mean size of blood vessels formed in implants

Figue. 10H showed the mean size of blood vessels in scaffolds with different interconnection sizes. It was noticed that blood vessel diameters increased within the first 4 weeks in all samples and then became stabilized. There was no significant change in new blood vessel diameters from 4 to 12 weeks. Although the blood vessels' mean size increased with increasing interconnection size, no significant difference was shown between scaffolds with interconnection sizes of 120 and 150 μm, and those between 120 and 100 μm at both time points (p > 0.05).

#### Newly formed blood vessel volume fractions in scaffolds

The volume percentage of blood vessels is affected by their amount and size, so this parameter can represent the extent of the implanted scaffolds' vascularization. The increased percentage of blood vessel volume out of scaffold volume (BV/SV) with time was observed in all three kinds of scaffolds. The newly formed blood vessels' ingrowths were observed in both kinds of implants (scaffolds with interconnection sizes of 100 and 120 μm) at 4 and 12 weeks after implantation (Fig. 10A–B, D–E), but no significant difference in new blood vessel volume fractions was observed (p > 0.05). At 12 weeks, it was clear that the BV/SV (%) in scaffolds with larger diameter interconnections (150 μm, 13.5 ± 0.58%) was remarkably higher than that of the samples with smaller diameter interconnections (5.76 ± 0.52% for 100 and 6.5 ± 0.63% for 120 μm, both p < 0.05). Specifically, the 150 μm group exhibited more obvious new blood vessel ingrowths into the implants (Fig. 10A–F, I). The above results indicate positive effect from scaffolds with a 150 μm interconnection size on neovascularization *in vivo*.

## Discussion

Three-dimensional porous biomaterials have been extensively used as scaffolds in the field of tissue engineering for *in vitro* study of cell–scaffold interactions and tissue synthesis and *in vivo* study of induced tissue and organ regeneration. Preparing an ideal biomaterial as a scaffold is a key procedure in neoangiogenesis and tissue engineering. Materials' porous architectural characteristics have a profound effect on vascularization post-implantation, which provides the basis for cell survival and tissue growth in porous biomaterials. Pore and interconnection between adjacent pores as two critical biomaterial structural parameters have a strong impact on cell processes, and previous studies have shown the upper limit of pore size for vascularization is 400 mm^24^,^25^. However, interconnection size's effect on vascularization and its related mechanisms have not been systematically investigated.

By using assembled organic microspheres as templates combined with a casting technique, we can not only precisely control biomaterials' internal pore structures, but also unequally control the macropore and interconnection sizes of porous scaffolds in different dimensions, respectively. To investigate the effect of interconnection size on neoangiogenesis, we used this technique to produce a series of porous β-TCPs with the same 300–400 μm pore sizes and variable interconnection sizes of 100, 120 and 150 μm. Each scaffold had a consistent pore structure and showed no significant variation in mean pore size, structure, or alignment at separate points within the scaffold, indicating the homogeneity of the scaffolds produced (Table 1). The results of micro-CT (Fig. 1) and SEM (Fig. 2) examination showed that the mean size of pores and prepared porous β-TCP interconnection sizes were accurately controlled in the theoretical scope. The scaffolds with high porosity and accurately controlled parameters overcome the limitations, due to imprecise structural characteristics, and guarantee the rationality of present study concerning interconnection size on blood vessel formation in β-TCP bioceramics.

In this study, we investigated the behavior of HUVECs, which play an important role in angiogenesis, on each kind of porous β-TCP scaffold, including cell attachment, proliferation, migration and cell-specific marker expression. We demonstrated that the scaffold with a 150 μm interconnection size not only increased cell viability, daily glucose consumption and cell attachment of HUVECs but also increased the expressions of PECAM-1 and VEGF, promoting the neovascularization of scaffolds implanted in rabbits' femoral condyles. Furthermore, this interconnection-induced improved vascularization behavior may be attributed to, at least partially, activating the PI3K/Akt signaling pathway.

The morphological observations using SEM (Fig. 3) indicated that cells could attach and proliferate well on all kinds of highly interconnected porous scaffolds, however, the specimens with interconnection sizes of 100 and 120 μm showed that the cells proliferated sufficiently only in macropore edges, whereas only a few cells survived in the inner macropore surface and grew into the inner part of the scaffold through the interconnection pores (Fig. 3 A, B, D, E). This mass transfer limitation has been resolved by increasing interconnection sizes to 150 μm (Fig. 3 C, F). In scaffolds with an interconnection size 150 μm, the dense and large-pieced endothelial cell layer was uniformly distributed on the whole surface of macropores, gathered around connecting holes, and grew into the inner part of the scaffold. These SEM results also indicated that a higher cell number was present in the scaffolds with a 150 μm interconnection size. The continued cell proliferation in these scaffolds has been verified by different methods in this study. Measurements of cell viability and daily glucose consumption revealed increased cell proliferation on scaffolds with a 150 μm interconnection size compared to the 100 and 120 μm sizes (Fig. 4, 5). The reason might lie in that scaffolds with a 150 μm interconnection size could promote cell junctions and aggregations, and help cells more easily gather around connecting holes, which leads to more cells growing through collecting holes. Thus, more space will be provided for cell proliferation and there will be higher cell numbers and glucose consumption in this kind of scaffold.

We clearly observed endothelial cell cytoskeletal organization on the scaffolds in Fig. 8. Cytoskeletal proteins such as actin have been used to illustrate the spreading and attachment of cells on scaffolds^26^. Our F-actin labeling results showed that HUVECs seeded on scaffolds with an interconnection size of 150 μm exhibited uniform and homogenous actin distribution in cytoplasm. This indicates that the cells cultured on scaffolds with a 150 μm interconnection size have stronger ability of migration. What's more, abundant F-actin was distributed in the surface of whole macropores, while a small amount of actin fibers existed in the inner surface of macropores in scaffolds with interconnection sizes of 100 and 120 μm (Fig. 8D–F). These are consistent with the results obtained from cell viability, glucose consumption measurements and morphological observations by SEM. Few reports demonstrated the direct activation of cell proliferation, migration and attachment on scaffolds with different interconnection sizes. Surprisingly, all these results indicated that scaffolds with a 150 μm interconnection size have promotional effect on cell proliferation, migration and attachment on scaffolds.

For the purpose of investigating the angiogenic potential of cells seeded on the β-TCP scaffolds with different interconnection sizes. Cell adhesion molecule PECAM-1 expression at the cell–cell interface could be used to indicate the microcapillary-like structure or lumina. It is a 130-kDa trans-membrane glycoprotein member of the Ig super-family that is expressed in endothelial cells^27^. PECAM-1 expressed by HUVECs is known to be crucial for vessel formation and maintenance^28^. Our results showed that, in the 150 μm group, strong PECAM-1 expression can be clearly observed on the scaffolds (Fig. 6A). Some elongated networks, but no obvious lumina, were observed during this experimental period. That might because monocultured HUVECs were used in our study. The ability of HVUECs to form lumina may depend on the properties of the extracellular matrix, which can affect the migration of HUVECs^29^. Co-culturing endothelial cells with bone-forming cells may facilitate the tube formation *in vitro* on scaffolds since the bone-forming cells and HUVECs can produce cytokines and angiogenic growth factors^30^,^31^. Unlike the continuous PECAM-1 expression by the HUVECs seeded on scaffolds with a 150 μm interconnection size, a lower, scattered PECAM-1 expression can be observed in the 100 and 120 μm groups. This is probably due to the decreased HUVEC population found by MTT and SEM and the decreased HUVEC migration founded by Immunofluorescent F-actin staining. The decreased HUVEC population and decreased HUVEC migration in scaffolds with 100 and 120 μm interconnevtion sizes could separate individual HUVECs, leading to the inability of HUVECs to contact and form a microcapillary-like structure. The PECAM-1 ELISA results also indicated that the PECAM-1 concentration in the 150 μm group was significantly better than in the 100 and 120 μm groups (Fig. 6B). The bioactivity of scaffolds to induce angiogenesis was usually evaluated by assessing the ability of scaffolds to stimulate the release of angiogenic growth factors from endothelial cells. VEGF, as a critical regulator in physiological angiogenesis, is one of the most potent angiogenesis inducers^32^. VEGF expression is usually up-regulated during the early phase of fracture repair to induce angiogenesis. Therefore, the expression of VEGF has become an important indicator of a scaffold's ability to induce angiogenesis. In our study, we found the magnitude of VEGF secretion correlated with interconnection sizes when HUVECs were cultured on scaffolds, and an evident increase in VEGF content was detected in scaffold with a 150 μm interconnection size (Fig. 7B). This indicates that, although in the HUVEC monoculture, different interconnection sizes could influence VEGF expression *in vitro*. These results *in vitro* demonstrated that scaffolds with a 150 μm interconnection size may stimulate angiogenesis by influencing HUVECs, so it is considered to be angiogenic and could be used to test angiogenesis-inducing abilities *in vivo*.

Several strategies have been suggested to accelerate angiogenesis, including modification of the scaffold design, localized potent angiogenic inductive factor delivery, and prevascularization^33^,^34^. Biomaterial-based strategies for enhancing angiogenesis offer some advantages compared to other approaches, such as favorable controllability, decreased complication rate, and reduced cost. Previous studies have shown that appropriate internal structure of scaffold could stimulate angiogenesis^14^,^15^,^16^,^17^. However, the mechanism of the stimulation process is still unclear. Although the PI3-kinase/Akt/eNOS pathway has been demonstrated to exert a critical effect on angiogenesis in adult organisms while actively promoting endothelial repair and regeneration^35^,^36^, few reports demonstrate the direct implication of PI3K/Akt/eNOS pathway activation with the improvement of endothelial repair and regeneration in scaffolds with different interconnection sizes. Surprisingly, the supportive function of PI3K/Akt/eNOS pathway activation on HUVEC proliferation in scaffolds with a 150 μm interconnection size was discovered in our study. We demonstrated that the scaffolds with a 150 μm interconnection size markedly augmented Akt and eNOS phosphorylation, stimulated NO production, and enhanced HUVEC proliferation (Fig. 4, 7A, 9A, respectively), whereas LY-294002 inhibited the expression of p-eNOS, indicating that scaffolds with different interconnection sizes might modulate NO production through PI3K/Akt signaling then stimulate angiogenesis (Fig. 9B).

Our results demonstrated that the scaffolds with a 150 μm interconnection size are capable of promoting HUVEC proliferation, which was further supported by the *in vivo* study (Fig. 10). From 4 weeks post-operation, the area and volume percentage of blood vessels in the 150 μm group began to significantly increase compared to the 100 and 120 μm groups, whereas the increase in interconnection size did not affect blood vessel diameter at the two time points (Fig. 10G–I). Twelve weeks post-operation, capillaries infiltrated the central macropores of all implants and capillaries in the scaffold with a 150 μm interconnection size were more abundant, large, and mature than those in the scaffolds with interconnection sizes of 100 and 120 μm (Fig. 10D–F). From the above results, we can see that the scale of interconnections appeared to limit the blood vessels running through the adjacent pores. This finding is consistent with the work of Mastrogiacomo *et al*., who thought that the interconnection pathway could limit the bioceramic vascularization, representing a bottleneck for blood vessel invasion^12^. This phenomenon is understandable, as the macropores mainly provide the space for blood vessel growth, whereas the interconnections function as the doorway for blood vessel ingrowth, so that their size determines the area and number of blood vessels allowed to grow into the inner part of scaffold. It is thusly evident that the interconnection is very important for blood vessel formation in the scaffold. This was also proved by Fig. 10D. Although there are larger capillaries on the bottom of the peripheral region, these blood vessels could not infiltrate into the central macropores of scaffolds with an interconnection size of 100 μm. By evaluating the volume percentage of newly formed blood vessels, we dynamically observed the extent of vascularization in the implanted scaffolds by micro-CT at two time points. The micro-CT results confirmed our *in-vitro* experiment conclusions in this study. From the results obtained, we noticed an important effect of interconnection size on the extent of vascularization in biomaterial. The extent of vascularization in scaffolds continuously and significantly increased with increasing interconnection size. What's more, while the area and volume percentage of the blood vessels significantly increased with increasing interconnection size, the increased interconnection did not affect blood vessel diameter, which may indicate that this promoting effect was realized through making new blood vessels sprout from old ones and lead to the relative increase in the number of blood vessels.

## Conclusion

The present study quantitatively demonstrated that interconnection size plays an important role in the vascularization of β-TCP with accurately controlled porous structures. In the absence of extra angiogenesis-inducing additives, the *in vitro* results indicated that scaffolds with a 150 μm interconnection size may stimulate angiogenesis by enhancing HUVEC proliferation, migration and increasing PECAM-1 and VEGF expressions. We gained deep insight into the mechanisms associated with interconnection size on neovascularization by uncovering the role of the PI3k/Akt/eNOS signaling pathway in this process. The *in vivo* study in a rabbit femur defect model showed that the interconnection was functioning as the doorway for blood vessel ingrowth. It may attract clinical interest concerning the modification of porous structures and biofunctionalization targeting at the PI3k/Akt pathway to boast better blood vessel ingrowth. Furthermore, these results are expected to enrich our knowledge about neovascularization under different inner structures and provide a strategy involving scaffold with interconnection size of 150 μm to improve the vascularization of implants.

## Methods

### Porous β-TCP bioceramic fabrication

An organic skeleton constituting PMMA balls were first prepared to develop a porous structure. We combined identical PMMA balls together as a porogen agent using a chemical forming treatment. This treatment established a connection between PMMA balls by a chemical superficial dissolution of the individual beads. This reaction was accompanied by significant organic skeleton shrinkage, which was correlated with the interconnection size between beads. To measure the organic scaffold shrinkage caused by the PMMA ball coalescence, a piston precisely sliding in the metal mould was put into contact with the ball bed surface in its highest part. An electronic displacement sensor was positioned on the top of piston, with a resolution of one micrometer (Tesa Digico 11 Tesa, France), which records the polymeric material movement. For different shrinkages, the chemical dissolution or bridging between balls were stopped by water action. Stoichiometric β-TCP powders were synthesized by the reaction between a diammonium phosphate solution (NH_4_)_2_HPO_4_ and a calcium nitrate solution Ca(NO_3_)_2_·4H_2_O. We poured the β-TCP aqueous slurries into a plaster mold containing the polymeric frame and filled the voids between polymeric particles to obtain green disks with 15 mm diameters and a 4 mm thickness. To create macroporosity within the ceramics, the PMMA was eliminated by a debinding treatment that was carried out at a low temperature after drying of the sample in the plaster mold. Heating the polymer at 220°C for 30 h allowed a large amount to be burned. The residual organic was then eliminated by heating it at 400°C for 5 h. After this debinding treatment, samples were sintered at 1100°C for 3 h to consolidate the ceramics. After sintering, the samples were cooled to room temperature in the furnace. The β-TCP disks were sterilized with ethylene oxide and sealed in a sterile package until use.

The porous bioceramic scaffold pore dimension was controlled by the initial choice of size of PMMA balls constituting the organic skeleton. The interconnection size was adjusted by the ball pile's shrinkage amplitude during the chemical forming treatment. Using this technique, we prepared three sets of disk-type porous β-TCP with the same macropore sizes and different interconnections (Table 1). Three kinds of scaffolds possessed the same 300–400 μm pore size, and the interconnection varied from 100 to 150 μm. The porous structures were observed by Scanning electron microscope (SEM, S-4800, Hitachi, Japan) and micro-CT (explore Locus SP, GE, USA). The mean macropore diameter and porous bioceramic interconnection sizes were calculated using the analysis software package (Microview ABA 2.1.2, GE, USA), and the porosity was determined by the Archimedian method^37^,^38^,^39^.

### *In vitro* study

#### Cell preparation and culture

All experimental protocols were conducted according to the guidelines approved by the Xijing Hospital affiliated to The Forth Military Medical University in Xi'an, China. Human umbilical vein endothelial cells were isolated and cultured as described previously, with written informed consent from the donors^40^. The umbilical vein was digested with 0.1% collagenase I (Sigma, St. Louis, MO, USA) for 15 min at 37°C. Subsequently, the cells were collected and cultured in ECM (Sciencell, San Diego, US) at 37°C in a humidified atmosphere of 5% CO_2_. Non-adherent cells were removed after 24 h. Cells were passaged when they reached an approximately 80% confluence. The HUVECs in the third passage were seeded on scaffolds with different interconnection sizes of 100, 120 and 150 μm at a density of 6 × 10^5^ cells/ml. Then the cell/scaffold composites were further incubated for 3, 7 and 14 days. Culture medium was changed every 2 days and media samples were collected at each media change. Nine cell/scaffold composites were cultured for each time point, and three different kinds of scaffolds (interconnection size 100, 120, 150 μm) had three samples, respectively.

### HUVEC morphologies on the β-TCP scaffolds

Scanning electron microscope was used to evaluate the morphological HUVEC appearance on β-TCP scaffolds. Briefly, scaffolds were washed twice with PBS and fixed in 2.5% glutaraldehyde at 4°C overnight, followed by graded dehydration using a series of ethanol solutions (generally 70%, 80%, 90% and 100%). Samples were then dried in a hood and sputtered with gold before observation under an SEM at 20 kV.

### Cell viability assays

Cell viability was evaluated by (3-(4, 5-dimethylthiazol-2-yl)-2, 5-diphenyltetrazolium bromide (MTT) colorimetric assay^41^. In brief, each scaffold was rinsed with PBS and then MTT solution (0.5 mg/ml in PBS) was added. After 4 h of incubation at 37°C, MTT solution was removed and 5 ml dimethyl sulfoxide (DMSO, Sigma) added to lyse the cells and solubilize the purple formazan crystals formed by vital cells. After 20 min of incubation, the samples were rinsed multiple times with DMSO to ensure a homogeneous dye solution. The optical density (OD) was measured using a spectrophotometric at 570 nm. Three samples were analyzed for each group.

### Glucose consumption

The cellular utilization of D-glucose was analyzed via spectrometry according to protocol developed by Roche (D-glucose UV-method, Roche, Germany). Samples were collected at each medium change and diluted appropriately to stay within the assay detection range. The assay was performed following the instructions provided by the kit manufacturer, and the absorbance of each cuvette at 505 nm was measured on a spectrophotometer. The fresh complete medium and D-glucose control solution were also processed and served as the reference. Finally, glucose concentration was quantified based on the absorbance difference, and glucose consumption was expressed as the average daily glucose reduction in the medium.

### Immunofluorescent HUVEC PECAM-1staining on the β-TCP scaffolds

A platelet-endothelial cell adhesion molecule (PECAM-1 or CD31) is an endothelial-specific adhesion protein and specific HUVEC marker. It was assessed by immunefluorescent staining. The effect of each β-TCP scaffold on expression of PECAM-1 was investigated at 7 and 14 days. Samples were rinsed and fixed in 4% paraformaldehyde solution (PFA) for 15 min at room temperature. The fixed cell/scaffold was placed in 3% bovine serum albumin (BSA) blocking buffer for 1 h. After incubating with rabbit anti-human CD31 primary antibody (1:50, Abcam) overnight at 4°C, the cell/scaffold was incubated in an anti-rabbit secondary antibody Alexa Fluor ®594 (1:1000; 2 μg ml^−1^, Invitrogen) for 1 h at room temperature. Then the cell nucleus was counterstained with DAPI solution (5 μg ml^−1^) for 3 min. The sample was mounted and imaged using confocal laser scanning microscopy (CLSM).

### Visualization of F-actin

F-actin was stained with rhodamine-phalloidin to assess cytoskeletal organization on the β-TCP scaffold. After 7 days of incubation, the cell/scaffold was fixed by 4% PFA for 15 min at room temperature and further permeabilized in 0.1% Triton X-100 for 5 min. After blocking non-specific antibody binding with 3.0% BSA for 30 min, the sample was stained with 100 nM rhodamine-phalloidin working solution (Cytoskeleton Inc., USA) at room temperature for 2 h and counter stained with DAPI solution (5 μg ml^−1^) for 10 min. The sample was then mounted and imaged using CLSM.

### Enzyme-linked immunosorbent assay (ELISA)

PECAM-1 and vascular endothelial growth factor (VEGF) concentrations in the culture mediums secreted from endothelial cells seeded on scaffolds were evaluated using ELISA. Samples were collected at each medium change. Supernatant liquid was collected after centrifugation and then stored at −20°C until protein quantification. The PECAM-1 enzyme-linked immunosorbent assay (ELISA) kit (Bender, Burlingham, CA, USA) was used to determine PECAM-1 content following the manufacturer's instructions. VEGF expression was quantified using ELISA kits (R&D Systems, Minneapolis, MN, USA) according to the manufacturer's instructions. The PECAM-1 and VEGF concentrations were determined by correlation with a standard curve and further normalized with the total cellular protein content, as previously described^42^. Three samples were analyzed for each group.

### Intracellular NO measurement

The production of NO was determined by measuring the nitrite level with the Griess reagent. We cultured HUVECs on scaffolds for 7 days with or without 30 μM LY-294002 (PI3K inhibitor), collected proteins from whole cell lysates, and quantified nitrite concentration using a NO Detection Kit (Jiancheng Technology, Nanjing, China) according to the manufacturer's instructions.

### Western blot analysis

After culturing for 7 days, HUVECs on the different β-TCP scaffolds were digested with 0.5% pancreatic enzyme and rinsed with PBS. Cells were lysed with cold RIPA buffer for 10 min, and the lysates were centrifuged at 12,000 rpm for 20 min at 4°C to pellet the cell debris. An aliquot of each lysate was taken out to measure protein concentration using a BCA protein assay kit (Thermo Scientific, Rockford, IL, USA). Five μg proteins in 2 × Laemmli loading buffer were heated at 95°C for 5 min and separated on 4–15% Mini-Protean TGXTM gels (BIO-RAD). Each lane was loaded with equal protein amounts. A Spectra™ multicolor broad range protein ladder (Fermentas) ran in parallel lanes. After electrophoresis, a PVDF membrane (Millipore, Billerica, MA) was used to transfer the proteins from gels in a buffer (192 mM glycine, 25 mM Tris, and 20% v/v methanol (pH 8.3)) and then blocked with 5% nonfat dry milk in TBST for 1 h. The membrane was incubated overnight at 4°C with p-Akt, Akt, p-eNOS, eNOS (1:500, rabbit polyclonal antibodies, CST, USA) and β-actin (1:1000, rabbit monoclonal antibodies, CST, USA), then washed three times in Tris-buffered saline (TBS) containing 0.05% Tween-20 (TBST) prior to 1 h of incubation with appropriate horseradish peroxidase-conjugated secondary antibodies at 37°C. The immunoreactive bands were visualized via the ECL chemiluminescence reagent (Millipore, USA).

### *In vivo* study

#### Scaffolds implantation in rabbit femoral defects

Eighteen healthy New Zealand white rabbits with an average weight of 3 ± 0.5 kg were randomly divided into three groups according to the porous β-TCP scaffolds with different interconnection sizes. The animals were housed in cages, in a sterile animal house, with free access to food and water. The scaffolds with interconnection sizes 100, 120 and 150 μm were respectively implanted into the bilateral femora of one rabbit, with a total of six samples in each time group for each material group. At each time point, six samples were collected for micro-CT to detect neovascularization. The rabbits were operated on under anesthesia by intravenously injecting 0.5 mg/kg of Acepromazine Calmivet-Vetoquinol and 10 mg/kg of Ketamine. Under rigorous sterile conditions, a critically sized defect was made in femoral condyles using an implant drill, which was orientated perpendicular to the longitudinal and sagittal femur axes^43^,^44^,^45^,^46^. Porous scaffolds were implanted into the defects, and the rabbits were sacrificed at 4 and 12 weeks after surgery.

The animal experiment was approved by the Fourth Military Medical University Committee on Animal Care and carried out at the Laboratory for Animal Research of Research Institute of Orthopaedics in XiJing Hospital affiliated to the Fourth Military Medical University in China. All experiments involving animals were carried out in accordance with the approved guidelines.

### Angiography

At 4 and 12 week time points, we fixed rabbits in supine position after general anesthesia with an intramuscular injection of a mixture of Acepromazine Calmivet-Vetoquinol (0.5 mg/kg) and Ketamine (10 mg/kg). The skin was sterilized with povidone-isodine solution and a 5 cm middle abdominal incision was made to expose the abdominal aorta and inferior vena cava. After abdominal aorta intubation, the inferior vena cava was cut. The rabbit was perfused by heparin and physiological saline (50 IU/ml) until its lower limb was empty of blood. Then, 4% paraformaldehyde was infused to the abdominal aorta. After the animals were euthanized, the 60 ml Microfil contrast agent was infused with a fixed-dose (100 ml/h) rate. When finished, the entrance and exit were closed. After being stored overnight at 4°C, specimens containing the implants and surrounding bone tissue were collected from bilateral femoral condyles and fixed in 4% paraformaldehyde for one week for further Micro-CT analysis.

### Micro-CT analysis

The extracted specimens (n = 6 in each group) were fixed in 4% paraformaldehyde and decalcified in 15% EDTA for 3 weeks at 4°C, placed in the sample holder and scanned under the micro-CT. About 1600 1024^2^ pixel projections were acquired for each tomogram. The X-ray source voltage was set at 55 kVp and beam current at 114 μA using filtered Bremsstrahlung radiation. The scanning angular rotation was 360°, and the angular increment was 0.40°. The projections were reconstructed using a modified parallel Feldkamp algorithm, and segmented into binary images. The final reconstructed data were converted to 3-dimensional images. Specimens were constructed and evaluated using 3D analysis software. The blood vessel volume percentage versus the scaffold volume (BV/SV), blood vessel diameters and blood vessel areas were calculated using −600 to −200 threshold based on a Microfil blood vessel contrast agent.

### Statistical analysis

Data were expressed as means ± standard deviations. Statistically significant differences (p < 0.05) among the various groups were evaluated using one-way ANOVA. All statistical analysis was performed using SPSS 19.0 software.

## Author Contributions

X.X., Z.W. and J.L. conceived the experiments, X.X., D.L., P.G. and H.Z. performed the animal experimentation, X.X. and W.W. performed the *in vitro* experiments, L.G. and Y.Y. prepared the tables and figures, X.X., W.W. and Z.W. wrote the manuscript. All authors reviewed the manuscript.

## Figures and Tables

**Table 1:** The theoretical and measured pore sizes and interconnection sizes

No.	Theoretical pore size (μm)	Theoretical interconnections (μm)	The measured pore size (μm)	The measured interconnections (μm)	Porosity (%)
A	300-400	100	320.4 ± 12.3	104.53 ± 12.96	72.75 ± 1.04
B	300-400	120	315.3 ± 21.9	117.23 ± 13.28	74.90 ± 1.60
C	300-400	150	321.3 ± 35.4	149.97 ± 12.56	77.68 ± 2.53
